# Reduced cortical bone thickness increases stress and strain in the female femoral diaphysis analyzed by a CT-based finite element method: Implications for the anatomical background of fatigue fracture of the femur

**DOI:** 10.1016/j.bonr.2020.100733

**Published:** 2020-11-12

**Authors:** Daisuke Endo, Keiko Ogami-Takamura, Takeshi Imamura, Kazunobu Saiki, Kiyohito Murai, Keishi Okamoto, Toshiyuki Tsurumoto

**Affiliations:** a Department of Macroscopic Anatomy, Graduate School of Biomedical Science, Nagasaki University, 1-12-4 Sakamoto, Nagasaki, Nagasaki 852-8523, Japan; b Center of Cadaver Surgical Training, Nagasaki University School of Medicine, 1-12-4 Sakamoto, Nagasaki, Nagasaki 852-8523, Japan

**Keywords:** Femur, Biomechanical parameter, Diaphysis, Cortical bone, Morphological parameter, Atypical femoral fracture

## Abstract

The incidence of hip fractures is increasing in Japan and is high among women older than 70 years. While osteoporosis has been identified as one of the causative factors of fracture, atypical femoral fracture has emerged as a potential complication of bisphosphonate therapy. Atypical femoral fracture is prevalent among Asian women and has been attributed to morphological parameters. Age-related decreases in the morphological parameters of the femoral diaphysis, such as cortical bone thickness, cortical cross-sectional area, and the cortical index, were reported in Japanese women prior to bisphosphonate drugs being approved for treatment. Thus, in the present study, the relationships between biomechanical and morphological parameters were analyzed using a CT-based finite element method.

Finite element models were constructed from 44 femurs of Japanese women aged 31–87 years using CT data. Loading conditions were set as the single-leg configuration and biomechanical parameters, maximum and minimum principal stresses, Drucker-Prager equivalent stress, maximum and minimum strains, and strain energy density were calculated in 7 zones from the subtrochanteric region to distal diaphysis. Pearson's correlation coefficient test was performed to investigate relationships with morphological parameters.

While absolute stresses gradually decreased from the subtrochanteric region to distal diaphysis, absolute strains markedly declined in the proximal diaphysis and were maintained at the same levels as those in the distal regions. All types of stresses and minimum principal strain in the femoral diaphysis scored higher absolute values in the high-risk group (≥70 years, n = 28) than in the low-risk group (<70 years, n = 16) (p < 0.05). The distribution patterns of equivalent stress and strain energy density were similar to that of Young's modulus, except for the region of the linea aspera. All biomechanical parameters correlated with morphological parameters and correlation efficiencies, with the reciprocal of cortical bone thickness showing the strongest correlation.

The present results demonstrated that biomechanical parameters may be predicted by calculating the cortical bone thickness of femurs not treated with bisphosphonates. Furthermore, strain appeared to be repressed at a low level despite differences in stress intensities among the regions by bone remodeling. This remodeling is considered to be regulated by Wolff's law driven by equivalent stress and strain energy densities from the proximal to distal femur. The present results will promote further investigations on the contribution of morphological parameters in the femoral diaphysis to the onset of atypical femoral fracture.

## Highlights

• Stresses in the femoral diaphysis were higher in the high-risk group (≥70 years).
• The distributions of equivalent stress were similar to that of Young's modulus.
• All biomechanical parameters strongly correlated with the cortical bone thickness.

## 1 Introduction

Hip fractures are prevalent and a significant cause of disability and mortality. The incidence of hip fractures continually increased between 1987 and 2012 (Orimo et al., 2016), and the risk of fracture was found to be approximately 1.5–2-fold higher in males and females older than 70 years in Japan (Tamaki et al., 2019). Previous studies reported that the risk of hip fracture was higher in women than in men (Greenspan et al., 1998; Kanis et al., 2002; Sáez-López et al., 2017). Osteoporosis, an age-related degenerative disease characterized by low bone mass, the microarchitectural deterioration of bone tissues, and decreased bone strength, has been identified as one of the causative factors of fracture (Coughlan and Dockery, 2014) and is treated by drugs such as bisphosphonates. Although bisphosphonates increase bone mineral density in the proximal femur (Bone et al., 2004), atypical femoral fracture (AFF), a type of fatigue fracture in the subtrochanteric region or proximal diaphysis, has emerged as a potential complication of bisphosphonate therapy (Shane et al., 2014). The risk of fracture was previously shown to be 4–7-fold higher in 75-year-old women than in 45-year-old women even though bone mass was identical (Hui et al., 1988). Moreover, although a seven-fold difference was observed in their effects on bone mineral density, all types of anti-resorptive treatments for osteoporosis exhibited the same efficacy to reduce the risk of fracture (Burr, 2004). These findings suggest the existence of other risk factors for hip fracture. Since AFF is more prevalent in Asian women than in Caucasian women, the specific geometry of the femur in the former group may be a candidate risk factor for fracture (Marcano et al., 2014). Relationships were recently reported between the risk of AFF, the neck-shaft angle of the femur (Hagen et al., 2014), and bowing of the femoral shaft (Oh et al., 2014a; Starr et al., 2018; Haider et al., 2019). Furthermore, the site of AFF was shown to shift from the subtrochanteric to diaphyseal region with a larger lateral bowing angle (Hyodo et al., 2017; Kim et al., 2017). Cortical bone may also be a target for fracture prevention. Caucasian and black women in America are at a high risk of typical and AFF and have femurs with a smaller cortical mass relative to body size than men (Jepsen et al., 2015; Schlecht et al., 2015). Furthermore, the complete loss of trabecular bone only had a negligible effect on bone strength (Holzer et al., 2009). Thus, cortical bone strongly contributes to the strength of the femur. Imamura et al. focused on the morphology of the femoral diaphysis and, based on CT data, reported its cortical bone thickness, cross-sectional area (area of cortical bone in a cross-section), the cortical index, and periosteal border length in Japanese women and men before bisphosphonate drugs had been approved for treatment in order to obtain a more detailed understanding of the anatomical background of AFF (Imamura et al., 2019). In women, age-related decreases were observed in cortical bone thickness, cross-sectional area, and the cortical index, and significant differences were noted in these morphological parameters between a younger group (<70 years) and older group (≥70 years). These findings are consistent with the high risk of hip fracture in Japanese women older than 70 years described above and suggest a relationship between the risk of fracture and morphological changes in cortical bone. Therefore, we hypothesized that the risk of fracture in the femoral diaphysis may be predicted by morphological parameters and conducted the present study to elucidate the anatomical background of fatigue fracture of the femur. We investigated the involvement of age-related changes in cortical bone morphology in the biomechanical characteristics of the femoral diaphysis in Japanese women. The femur is a complex structure that comprises materials with heterogeneous element characteristics, such as density and Young's modules. Although difficulties are associated with analyzing the biomechanical characteristics of the femur, a three-dimensional femur model may be generated based on CT data, thereby allowing mechanical characteristics to be calculated non-invasively using a CT-based finite element method. Previous studies reported that the data obtained from a CT-based specimen-specific finite element analysis model of the femur were accurate and useful in the stance condition (Rybicki et al., 1972; Bessho et al., 2007; Miura et al., 2017). A finite element analysis has the ability to predict the risk of fracture of the femur more accurately than a bone mineral density analysis (Langton et al., 2009; Qasim et al., 2016). Keaveny previously reported that femoral strength calculated using a finite element analysis decreased in Caucasian women and men with age, and an additional increase was noted in the prevalence of low femoral strength in women than in men entering the seventh decade of life (Keaveny et al., 2010). This method also revealed that femoral geometries, such as the neck-shaft angle and femoral bowing, were related to tensile stress and strain and may be a risk marker for AFF (Oh et al., 2014b; Oh et al., 2017; Haider et al., 2018). Therefore, to investigate whether the risk of fracture may be predicted based on morphological parameters, the distribution of biomechanical parameters in the femoral diaphysis and their relationships with cortical bone thickness, cross-sectional area, and the cortical index were examined using a CT-based finite element method in the single-leg stance configuration.

## 2 Materials and methods

### 2.1 Materials

The same skeletons as those described in our previous study were used (Imamura et al., 2019); among skeletal specimens of modern Japanese stored at Nagasaki University, 44 right femurs from females aged 31–87 years (mean age, 68.3 years) were examined. They were obtained from cadavers provided to the Nagasaki University School of Medicine for anatomical dissection by medical students between the 1950s and 1970s; most were voluntarily donated and from anonymous subjects. The sex and exact age at death of all individuals were registered. After dissection, soft tissues were almost entirely removed to produce dry skeletal preparations. Femurs with obvious trauma or inflammatory joint diseases were excluded. They were kept in a dark room at room temperature until computed tomography (CT) imaging. Due to the year of death, none of the subjects had been treated with bisphosphonate drugs. All procedures performed in the present study were in accordance with the standards of the Ethics Committee of Nagasaki University Graduate School of Biomedical Sciences (approval number: 15033076) and with the 1964 Declaration of Helsinki and its later amendments or comparable ethical standards.

### 2.2 CT imaging and extraction of target images

**Fig. 1:**
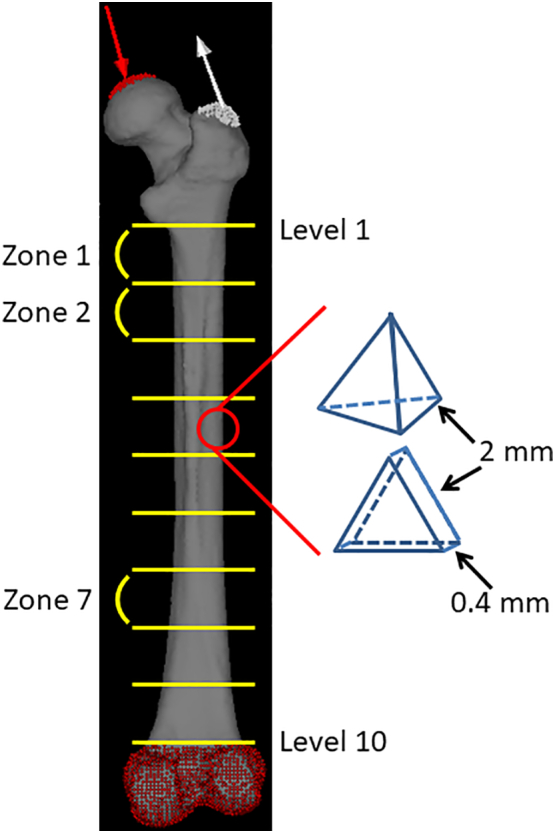
Diagram of reconstruction using the CT-based FE analysis. Three-dimensional FE models were constructed using 2-mm tetrahedral elements for the trabecular and inner cortical bones. The outer surface of cortical bone is represented by a three nodal-point shell element with a thickness of 0.4 mm. The loading direction in the single-leg stance configuration was indicated by red and white arrows. The construction was full fixation in all directions under the adductor tubercle. The range between the lower end of the lesser trochanter and adductor tubercle of each femur was divided into nine segments of equal lengths. Segments were labeled “zone 1” to “zone 9”, creating seven zones (from zones 1 to 7). Cross-sections, including both ends, were also labeled from top to bottom as “Level 1” to “Level 10”.

Full-length images of all examined femurs were obtained using clinical multislice CT (Activision 16, Toshiba Corp., Tokyo, Japan) (X-tube volume/current = 120 kV/100 mA, image matrix size; 512 × 512 pixels, slice thickness; 0.5 mm). This slice thickness resulted in an error of up to 0.6% in morphological parameters. Since plane resolutions of CT were assessed using a field size divided by 512, they were not constant and ranged between 0.150 and 0.205 mm (mean value, 0.176 mm). Bones were placed in a natural position with the posterior side down on the table of the imaging device. Data were saved in the Digital Imaging and Communication in Medicine (DICOM) format. The range between the lower end of the lesser trochanter and adductor tubercle of each femur was divided into nine segments of equal lengths. Segments were labeled “zone 1” to “zone 9”, and seven zones (from zones 1 to 7) were subsequently analyzed (Fig. 1). Cross-sections, including both ends, were also labeled from the top to bottom as “Level 1” to “Level 10” and the morphological values of both ends of every zone were averaged. The relationships between averaged morphological values and the biomechanical parameters of each zone were analyzed. The threshold values for the definition of the cortex were calculated as described in our previous study (Imamura et al., 2019), and were assessed as follows: (i) all of the matrixes for the ten levels were pasted into one Microsoft Excel sheet, and (ii) a histogram was created based on a frequency table of CT values to calculate the mean CT value for the first peak (i.e., approximately −1000; mainly indicating the CT value of the surrounding air) and the CT value for the second peak (i.e., indicating the CT value of the bone itself).

### 2.3 CT-based finite element analysis

FE models of the femurs were constructed using CT data from Mechanical Finder version 10.0 (Research Center of Computational Mechanics, Inc., Tokyo, Japan), which is software that reconstructs individual bone shapes and density distributions. A 3-dimensional FE model with 2-mm tetrahedral elements for the trabecular and inner cortical bones and 3 nodal-point shell elements with a thickness of 0.4 mm for the outer surface of cortical bone was constructed for each femur (Fig. 1). Each model consisted of approximately 450,000 elements. The mechanical properties of the bone, such as the specific density-elastic modulus, namely, Young's modulus, were assessed using CT density values based on the equations proposed by Keyak et al. (1998). Young's modulus of each tetrahedral element was calculated using the equations proposed by Keyak. Poisson's ratio was set to 0.4 as described previously by Dong and Guo (2004).

Based on previous studies (Huiskes et al., 1987; Herrera et al., 2007; Hirata et al., 2013; Ike et al., 2015), in the single leg stance configuration, loading conditions were set at 4 times their estimated body weights on the femoral head in a direction 15° proximal-medial from the femoral axis along the frontal plane and 0° along the horizontal plane for body-weight loading, and at 2 times their estimated body weights to the greater trochanter in a direction 20° distal-lateral from the femoral axis along the frontal plane and 0° along the horizontal plane for the abductor muscle force. The construction was full fixation in all directions under the adductor tubercle (Fig. 1). Their body weights were estimated by the femoral head breath (Ruff et al., 1997) (mean body weight, 52.9 kg). The maximum values of maximum principal stress, Drucker-Prager equivalent stress (calculated using equivalent von Mises stress, hydrostatic stress, and material constants (Drucker and Prager, 1952)), maximum principal strain, and strain energy density were calculated on each zone of the femoral diaphysis using these settings. The minimum values of minimum principal stress and strain were also calculated. Four models of different mesh sizes were created to perform the mesh sensitivity test; (a) 5 mm, (b) 4 mm, (c) 3 mm, and (d) 2 mm. The convergence test in the present study was used to calculate maximum principal stress in zone 1. Percent changes between (a) and (d), (b) and (d), and (c) and (d) were 9.8, 8.2, and 2.7%, respectively. Four models of different shell thicknesses were also created to perform the shell thickness sensitivity test: (a) 0.2 mm, (b) 0.3 mm, (c) 0.4 mm, and (d) 0.5 mm. The convergence test in the present study was used to calculate maximum principal stress in zone 1. Percent changes between (a) and (c), (b) and (c), and (d) and (c) were 7.0, 4.7, and 6.1%, respectively.

### 2.4 Statistical analysis

To confirm the standard distribution of each parameter, the goodness of fit test was performed. A repeated measures two-factor ANOVA was conducted to analyze differences in parameters between the low-risk (<70 years of age, n = 16) and high-risk (≥70 years of age, n = 28) groups based on significant differences in the rate of femoral fracture (Tamaki et al., 2019) and morphological parameters (Imamura et al., 2019) in these two groups. Pearson's correlation coefficient test was performed to examine the relationships among age and mechanical and morphological parameters. Regression lines were also drawn using a simple regression analysis. Differences in regression lines were analyzed with the F-test for linear regression. In the present study, expressions related to statistical analyses were defined as follows. The slope is ‘a’ in the equation for the linear regression line, y = ax + b. This value indicates the rate of change in y (the dependent variable) with changes in x (the independent variable). Absolute is a non-negative value without regard to its sign and is closely related to magnitude in a physiological context. Correlation coefficients are used to assess the relationship between two variables and the value between −1 (a strong negative relationship) and 1 (a strong positive relationship). Interaction effects occur when the effect of one variable depends on the value of another variable.

## 3 Results and discussion

The purpose of the present study was to clarify whether morphological parameters contribute to the risk of fracture in the femoral diaphysis. The parameters previously reported to decrease in women older than 70 years (Imamura et al., 2019) were herein examined in order to elucidate their relationships with biomechanical parameters calculated by a CT-based finite element method in the single-leg stance configuration.

### 3.1 Implications from the distribution of biomechanical parameters in the female femoral diaphysis

**Fig. 2:**
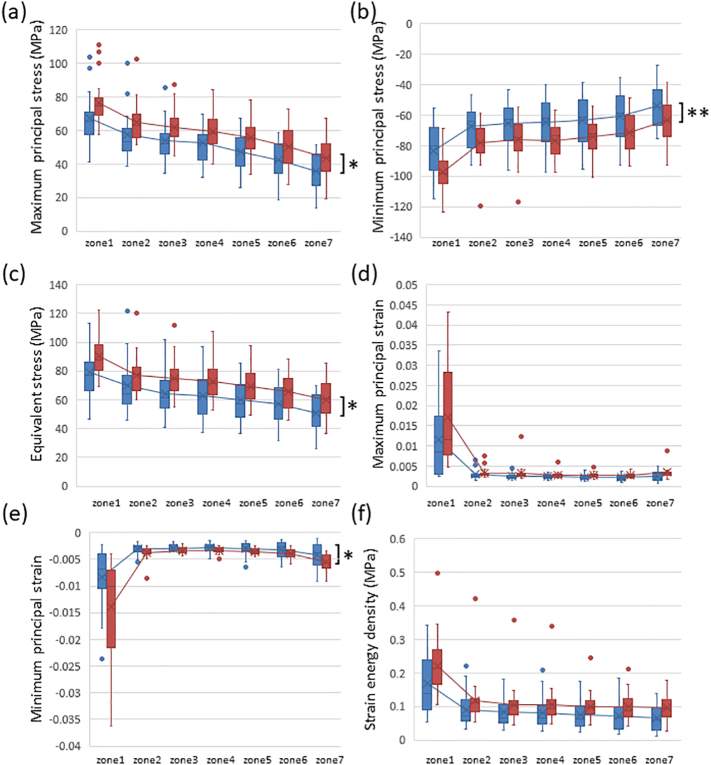
Box plots of comparisons of low-risk (blue box, <70 years old, n = 16) and high-risk (red box, ≥70 years old, n = 28) groups. Dots, -, and x show outliers, medians, and averages, respectively. (a) Maximum principal stress, (b) minimum principal stress, (c) equivalent stress, (d) maximum principal strain, (e) minimum principal strain, and (f) strain energy density. *: p < 0.05, **: p < 0.01.

**Supplementary Fig. 1:**
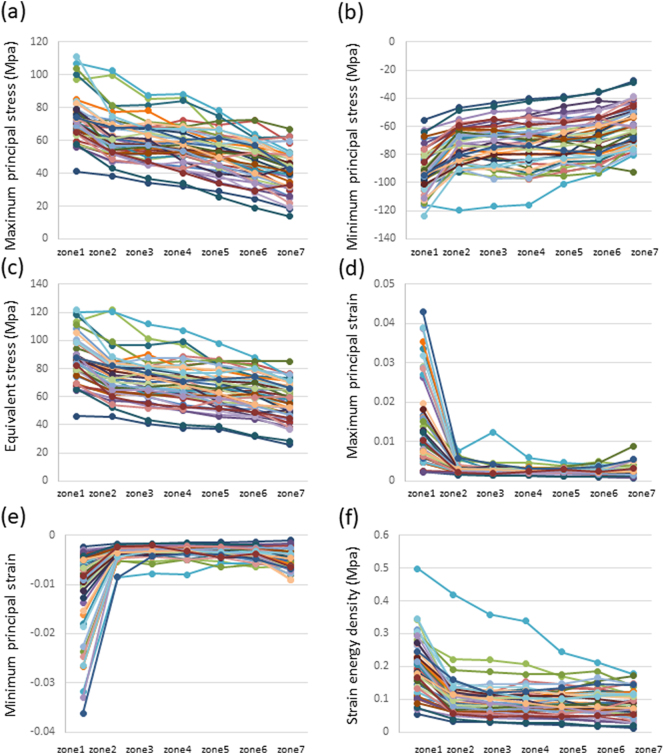
Biomechanical parameters of the femoral diaphysis in 44 females.

Among the 44 femurs examined, the absolute values of maximum principal stress, minimum principal stress, and equivalent stress linearly decreased from the subtrochanteric region to distal diaphysis (Supplementary Fig. 1). Although the absolute of every stress in zone 1 scored various values, they declined in parallel and the slopes of each femur from zones 1 to 7 were not significantly different. Minimum principal and equivalent stresses correlated with age in the subtrochanteric region and distal diaphysis, as shown in Table 1. Based on significant increases in the risk of femoral fracture (Tamaki et al., 2019), reductions in morphological parameter values (Imamura et al., 2019), and additional increases in the prevalence of low femoral strength in women after 70 years of age (Keaveny et al., 2010), the 44 femurs examined in the present study were divided into two groups: 16 low-risk (<70 years of age) and 28 high-risk (≥70 years of age). The medians and averages of the absolutes were higher in the high-risk group than in the low-risk group (Fig. 2). While the averages of all types of stresses and the minimum principal strain significantly differed between the low- and high-risk groups, comparisons of averages by a two-way ANOVA revealed no interaction effects. Therefore, age did not significantly affect the distribution of stress from the subtrochanteric region to the distal diaphysis of the femur. Correlation efficiencies with age were not high, indicating that age-related factors affected the broad ranges of absolute values.

On the other hand, strain values were the highest in the subtrochanteric region and similar in the shaft despite stress differences among the zones (Supplementary Fig. 1). Strains caused by different stress intensities may have been suppressed by bone remodeling in the femoral diaphysis. This result is consistent with Wolff's law, which states that bone in a healthy individual or animal will adapt to the loads under which it is placed (Wolff, 1986; Rubin and Lanyon, 1987). Strains and strain energy density also weakly correlated with age in the subtrochanteric region and distal diaphysis (Table 1) and the average minimum principal strain significantly differed between the low-risk and high-risk groups (Fig. 2). Similar to the results obtained for stresses, strain and strain energy density appeared to have been affected by age-related morphological factors.

**Fig. 3:**
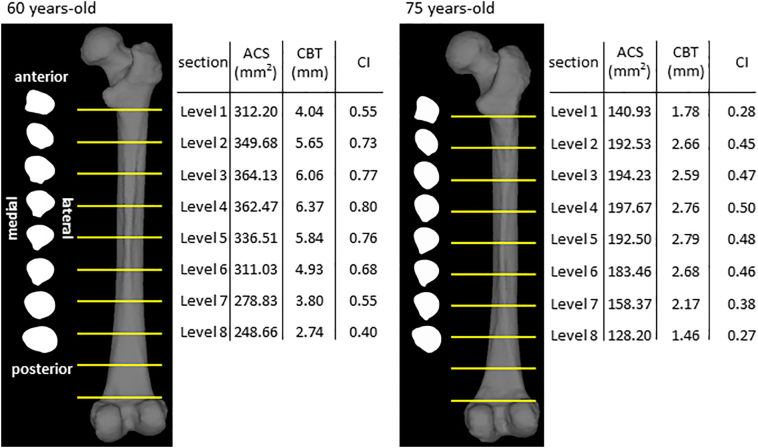
Morphological parameters in sections of the femoral diaphysis of 60- and 75-year-old females from levels 1 to 8 (same samples as in Fig. 4). The shapes of cross-sections for eight levels are shown. The values of the area of the cross-section (ACS), cortical bone thickness (CBT), and cortical index (CI) are shown in the table.

**Fig. 4:**
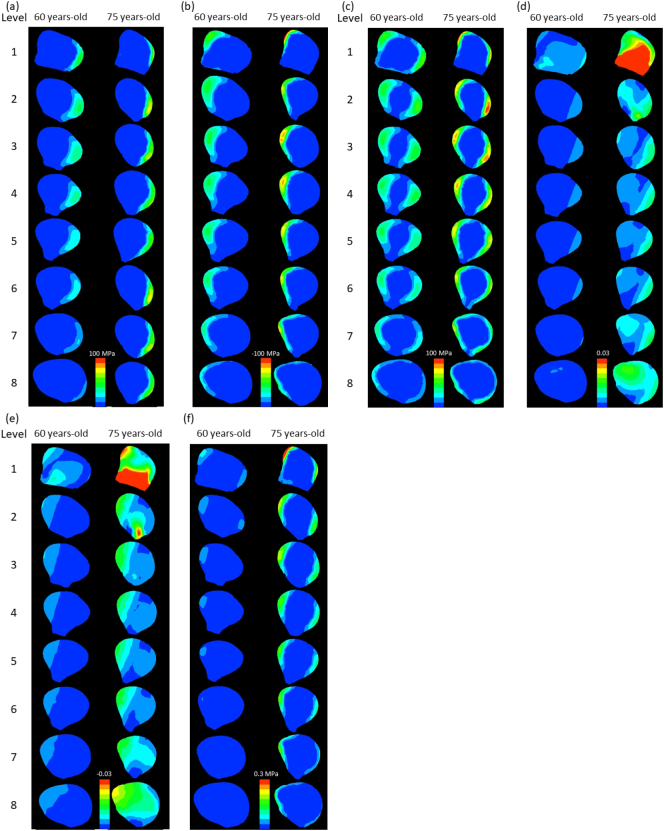
Distributions of biomechanical parameters in sections of the femoral diaphysis of 60- and 75-year-old females from levels 1 to 8. (a) Maximum principal stress, (b) minimum principal stress, (c) equivalent stress, (d) maximum principal strain, (e) minimum principal strain, and (f) strain energy density. In (a) and (c), the red color means >100 MPa and the blue color means 0 MPa. In (b), red means <−100 MPa. In (d), red means >0.01 and blue 0. In (e), red means <−0.01 and blue 0. In (f), red means >0.3 MPa and blue 0 MPa.

Fig. 3 shows the contours and morphological parameters of two femurs from the low- and high-risk groups. Low-risk femurs had a larger cortical bone thickness and cross-sectional area as well as a higher cortical index than high-risk femurs. The two-dimensional distribution of biomechanical parameters from levels 1 to 8 was investigated in all 44 femurs analyzed and representative images from the same samples in Fig. 3 were shown in Fig. 4. While age-related differences were observed in the absolute values of the parameters, distributions were similar in the femoral diaphysis from these 2 groups. Maximum principal stress was distributed in the posterolateral region and was stronger in the proximal than in the distal diaphysis. On the other hand, minimum principal stress was high in the anteromedial region. Stress in the tensile direction was distributed on the lateral side, while that in the compressive direction was on the medial side in accordance with the loading condition, tensile force of the abductor muscle on the greater trochanter, and compressive hip contact force on the femoral head. Regarding strain, maximum and minimum strains were both high in the posterior region of level 1 and low in the posterolateral and anteromedial regions from levels 3 to 7. The distribution pattern of equivalent stress was the sum of these two types of stresses. In the femur of a 75-year-old woman, both strains increased in the anteromedial region of level 7. Similar to the results obtained on equivalent stress, strain energy density was high in the anteromedial and posterolateral regions. Regarding strain, since maximum and minimum principal strains are caused by maximum and minimum principal stresses, respectively, the distribution patterns of strain and stress were similar after level 3. However, maximum and minimum principal strains were both high in the posterior region of levels 1 and 2, particularly in the femurs of 75-year-old individuals, and were attributed to a lower Young's modulus in the posterior region of levels 1 and 2 than that in the medial and lateral regions. A large strain caused by a low Young's modulus in the absence of high stress may be a high-risk factor for fracture. Although bisphosphonates suppress the activity of osteoclasts and prevent decreases in bone mineral density, bone remodeling is simultaneously retarded (Shane et al., 2014). As a consequence, if the distribution of stresses shifts from that of Young's modulus, a strong strain will be generated at the shifted regions. This gap caused by bisphosphonates may cause AFF.

**Table 1:** Correlation coefficients of biomechanical parameters and age from Zones 1 to 7.

	Maximum principal stress	Minimum principal stress	Equivalent stress	Maximum principal strain	Minimum principal strain	Strain energy density
Zone 1	n.s.	−0.45⁎⁎	0.36⁎	0.36⁎	−0.39⁎	0.31⁎
Zone 2	n.s.	n.s.	n.s.	n.s.	n.s.	n.s.
Zone 3	n.s.	n.s.	n.s.	n.s.	n.s.	n.s.
Zone 4	n.s.	−0.31⁎	n.s.	n.s.	n.s.	n.s.
Zone 5	n.s.	−0.33⁎	n.s.	n.s.	−0.33⁎	n.s.
Zone 6	n.s.	−0.31⁎	0.30⁎	n.s.	−0.33⁎	n.s.
Zone 7	n.s.	−0.34⁎	0.35⁎	0.43⁎⁎	−0.51⁎⁎	0.35⁎

n.s.: not significant.

⁎ p < 0.05.

⁎⁎ p < 0.01.

**Fig. 5:**
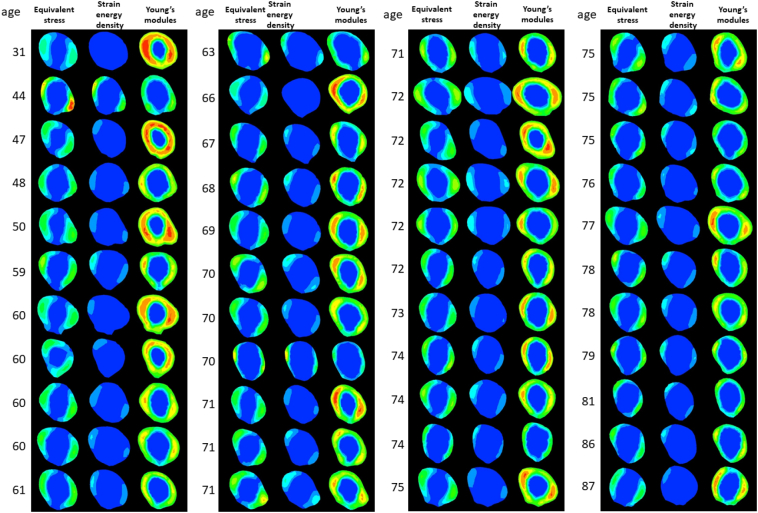
Distributions of equivalent stress, strain energy density, and Young's modulus in 44 femoral diaphysis sections at level 3 in ascending order of age. In the case of equivalent stress, the red color indicates >100 MPa and the blue color 0 MPa. In the case of strain energy density, the red color indicates >0.3 MPa and the blue color 0 MPa. In the case of Young's modulus, the red color indicates >30,000 MPa and the blue color 0 MPa.

**Supplementary Fig. 2:**
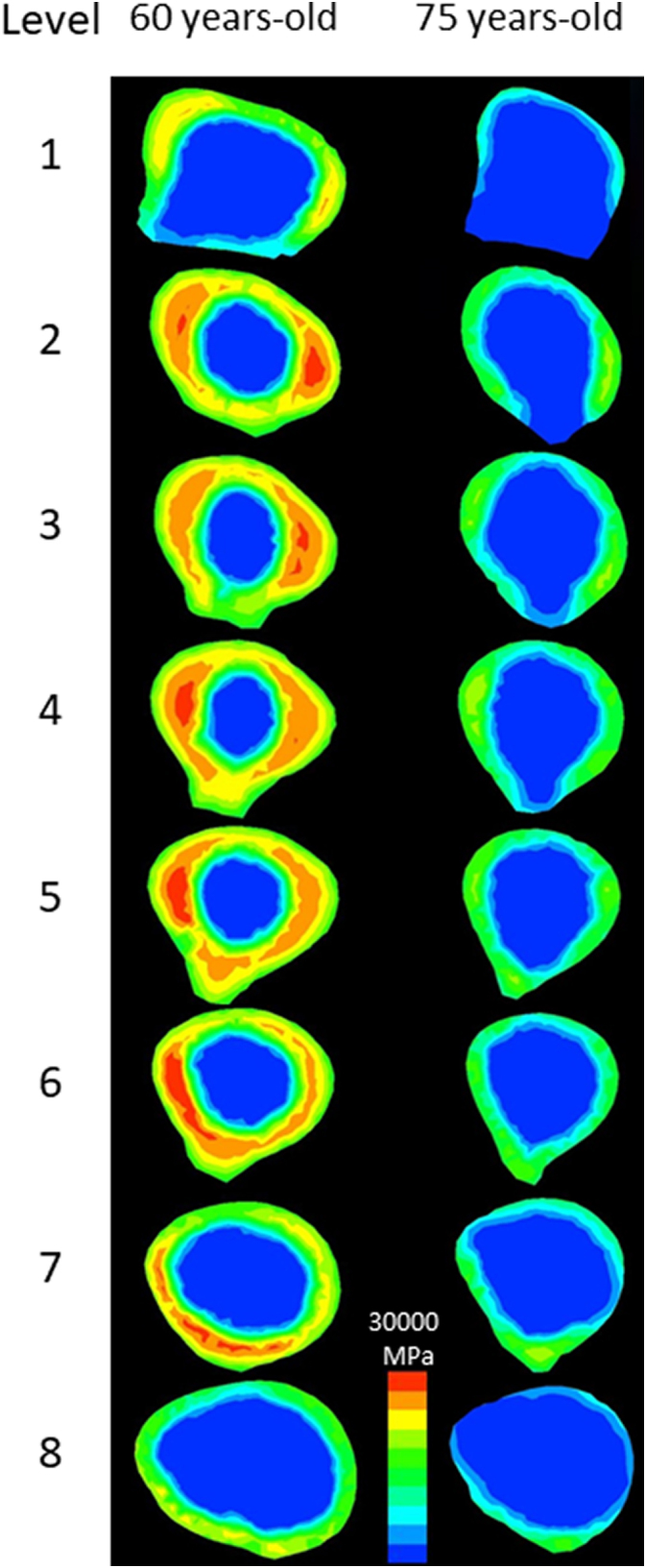
Distribution of Young's modulus in sections of the femoral diaphysis of 60- and 75-year-old females from levels 1 to 8. The red color means >30,000 MPa and the blue color means 0 MPa.

The distribution patterns of equivalent stress and strain energy densities were consistent with that of a high Young's modulus, except for the region of the linea aspera (Supplementary Fig. 2), in all 44 femurs analyzed. The images obtained at level 3, as a representative level, from all femurs were shown in Fig. 5. As discussed above, bone is remodeled in accordance with Wolff's law and candidates for its driving force were previously reported to be strain (Cowin and Hegedus, 1976), stress (Wolff, 1986), and strain energy density (Weinans et al., 1994). This overlap strongly suggests that equivalent stress and strain energy density are the driving forces of bone remodeling for elasticity. The high Young's modulus in the linea aspera cannot be explained by the present results. The present study only focused on the single-leg stance configuration and other daily activities, such as walking, and tension by the adductor muscle on this region may be responsible. Furthermore, the osteon population density was previously shown to be high in the lateral region of the femoral diaphysis and this is consistent with the distribution of maximum principal stress and strain observed in the present study (Gocha and Agnew, 2016). While these findings are mostly consistent with the present results, Matsuo et al. found that dark-type osteons for tensile stress were rich in the anterior and posterior regions, while bright-type osteons for compressive stress were abundant in the medial and lateral regions of the femoral diaphysis (Matsuo et al., 2019). The alignment of collagen fibers differs between dark- and bright-type osteons. These findings suggest that bone strength is regulated by various mechanisms and their driving forces differ.

### 3.2 Correlations between the area of cortical bone and biomechanical parameters

**Fig. 6:**
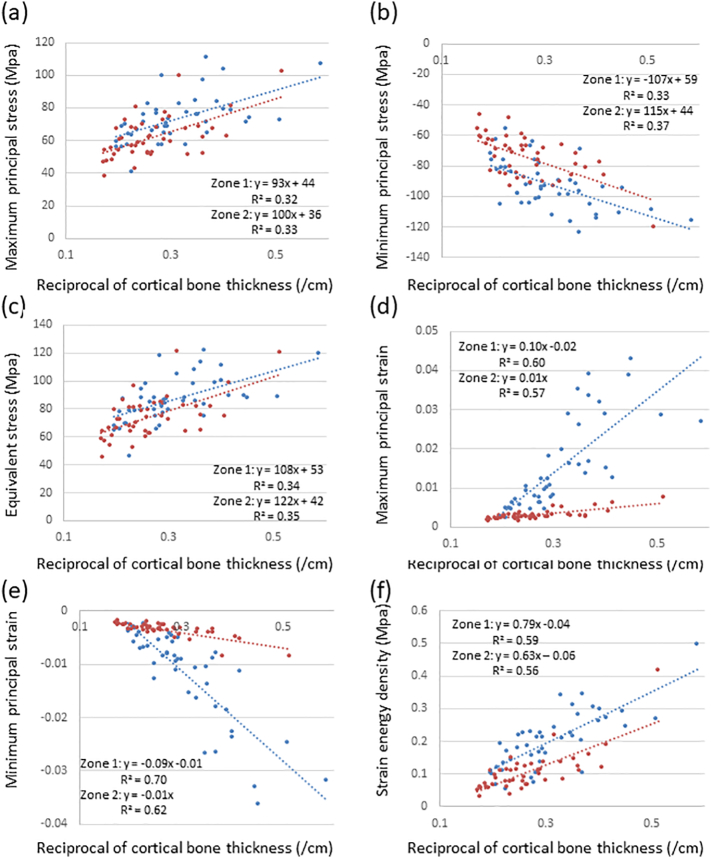
Scatter plots showing relationships between biomechanical parameters and the reciprocal of the cross-sectional area in Zone 1 and 2. (a) Maximum principal stress, (b) minimum principal stress, (c) equivalent stress, (d) maximum principal strain, (e) minimum principal strain, and (f) strain energy density.

Large deviations were observed in the biomechanical parameters of the 44 femurs analyzed. To elucidate the underlying causes, the relationships between these parameters and morphological characteristics were investigated. Since all parameters showed slight age-related increases in their absolutes and all types of stresses and minimum principal strain exhibited significant differences, the morphological parameters reported to decrease in the high-risk group, namely, cortical bone thickness, cross-sectional area, and the cortical index, were investigated in more detail (Imamura et al., 2019). Biomechanical parameters correlated with all morphological parameters in the femurs analyzed, except for maximum principal stress in the distal diaphysis (Table 2, Supplementary Tables 1, 2). Most of the correlation efficiencies were higher with the reciprocal of cortical bone thickness than those of other morphological parameters. Since stress inversely correlated with area, it is plausible that these coefficients were higher with the reciprocal of morphological parameters than with these parameters themselves. Coefficients of determination were more than 0.5 with strains and strain energy density, and half of the biomechanical parameters were predicted by the regression line with cortical bone thickness. As shown in Supplementary Table 3, cortical bone thickness was not independent of the cross-section area and cortical index. Thus, further studies are needed to identify other factors that may contribute to biomechanical parameters. Since the neck-shaft angle (Hagen et al., 2014) and bowing of the femoral shaft (Starr et al., 2018; Haider et al., 2019) were previously shown to influence the site of AFF and maximum principal stress (Oh et al., 2014b), these morphological parameters are strong candidates. Regarding stresses, as the relationship became weaker from the proximal to distal regions, factors other than cortical bone thickness appeared to be responsible in the distal femur. Although biomechanical parameters markedly differed in zones 1 and 2, scatter plots were similar and showed strong correlations with the reciprocal of cortical bone thickness. Despite the presence of higher stress in zone 1 than in zone 2, the slopes of regression lines for minimum principal and equivalent stresses were significantly higher in zone 2 than in zone 1 (p < 0.05 and 0.01, respectively) (Fig. 6). Stresses were more sensitively affected by cortical bone thickness in the proximal diaphysis than in the subtrochanteric region. If the femur cannot be correctly remodeled, a reduced cortical bone thickness plays an important role in the bone strength of the proximal diaphysis. On the other hand, the slopes of strains and strain energy density showed markedly higher values in zone 1 (p < 0.01) and small changes in cortical bone thickness resulted in large differences. In the subtrochanteric region, a reduced cortical bone thickness was identified as an important risk factor for fractures, even in femurs not treated with bisphosphonates.

**Table 2:** Correlation coefficients of biomechanical parameters and the reciprocal of cortical bone thickness from Zones 1 to 7.

	Maximum principal stress	Minimum principal stress	Equivalent stress	Maximum principal strain	Minimum principal strain	Strain energy density
Zone 1	0.56⁎⁎	−0.57⁎⁎	0.58⁎⁎	0.78⁎⁎	−0.84⁎⁎	0.77⁎⁎
Zone 2	0.57⁎⁎	−0.61⁎⁎	0.59⁎⁎	0.75⁎⁎	−0.79⁎⁎	0.75⁎⁎
Zone 3	0.44⁎⁎	−0.61⁎⁎	0.54⁎⁎	0.72⁎⁎	−0.81⁎⁎	0.74⁎⁎
Zone 4	0.32⁎	−0.51⁎⁎	0.45⁎⁎	0.58⁎⁎	−0.74⁎⁎	0.67⁎⁎
Zone 5	n.s.	−0.39⁎⁎	0.36⁎	0.56⁎⁎	−0.71⁎⁎	0.52⁎⁎
Zone 6	n.s.	−0.36⁎	0.33⁎	0.53⁎⁎	−0.66⁎⁎	0.51⁎⁎
Zone 7	n.s.	−0.31⁎	0.30⁎	0.57⁎⁎	−0.58⁎⁎	0.44⁎⁎

n.s.: not significant.

⁎ p < 0.05.

⁎⁎ p < 0.01.

### 3.3 Limitations of the present study

The present study had some limitations. Femurs without actual AFF were examined to clarify baseline conditions without any effect of bisphosphonates. Due to the nature of the anatomical skeletal collection, the population of samples was skewed towards older age ranges, we were unable to compare pre- and post-menopausal women, and it was not possible to investigate changes in the relationships between morphological and biomechanical parameters. Furthermore, invasive biomechanical tests were not permitted. Information on the physical status of each femur was also absent and estimated body weight was used for the loading condition. Morphological parameters other than the cross-sectional area, cortical bone thickness, and cortical index, such as bone curvature, were not quantified in the femurs used in the present study. Thus, it was not possible to examine the relationships between femoral bowing or the neck-shaft angle and mechanical parameters. Furthermore, configurations other than the single-leg stance were not analyzed.

## 4 Conclusions

Collectively, the present results demonstrated that biomechanical parameters, stresses, strains, and strain energy density may be predicted by calculating the cortical bone thickness of the femoral diaphysis not treated with bisphosphonates. A reduced cortical bone thickness caused strong stresses and strains in femoral shafts, particularly in the subtrochanteric region, and, thus, increased the risk of hip fracture. Strains appeared to be maintained at almost the same and low levels by Wolff's law driven by equivalent stress and strain energy density from the proximal to distal femur. The present study provides a basis for investigating how bisphosphonates affect morphology and the distributions of biomechanical parameters by analyzing CT data and using CT-based finite element methods. Moreover, by examining more morphological parameters, such as curvature and other loading configurations, including walking and falls, the relationship between the morphology of the femur and the risk of hip fracture may be more accurately predicted.

The following are the supplementary data related to this article.

Supplementary tables Image 1

## Ethical approval

All procedures performed in the present study were in accordance with the ethical standards of the Ethics Committee of Nagasaki University Graduate School of Biomedical Sciences (approval number: 15033076) and with the 1964 Declaration of Helsinki and its later amendments of comparable ethical standards.

## CRediT authorship contribution statement

D.E. and T.T. conceived and designed the study. T.I. and K.S. acquired CT data. D.E. and T.T. analyzed and interpreted data. D.E. and T.T. performed statistical analyses. D.E. and T.T. contributed to the drafting of the manuscript. K.O-T., K.M., and K.O. contributed to critical revisions of the manuscript and its approval for submission.

## Transparency document

Transparency document. Image 1

## Declaration of competing interest

The authors declare that there are no conflicts of interest regarding the publication of this manuscript.
