# Supplementary material for: Reduced cortical bone thickness increases stress and strain in the female femoral diaphysis analyzed by a CT-based finite element method: Implications for the anatomical background of fatigue fracture of the femur
Source: Bone Rep. 2020 Nov 12;13:100733. doi: 10.1016/j.bonr.2020.100733 (PMC7701322; doi:10.1016/j.bonr.2020.100733)
Supplement: Supplementary file 1 — Supplementary tables [file mmc1.docx]

|  | Maximum  principal  stress | Minimum  principal  stress | Equivalent  stress | Maximum  principal  strain | Minimum  principal  strain | Strain  energy  density |
| --- | --- | --- | --- | --- | --- | --- |
| zone 1 | 0.61** | -0.56** | 0.59** | 0.76** | -0.82** | 0.72** |
| zone 2 | 0.56** | -0.58** | 0.59** | 0.72** | -0.75** | 0.70** |
| zone 3 | 0.43** | -0.60** | 0.52** | 0.62** | -0.79** | 0.71** |
| zone 4 | 0.33* | -0.52** | 0.46** | 0.55** | -0.70** | 0.65** |
| zone 5 | n.s. | -0.43** | 0.38* | 0.50** | -0.72** | 0.56** |
| zone 6 | n.s. | -0.43** | 0.37* | 0.51** | -0.62** | 0.57** |
| zone 7 | n.s. | -0.35* | 0.33* | 0.52** | -0.52** | 0.50** |

Supplementary Table 1. Correlation coefficients of biomechanical parameters and

The reciprocal of the area of the cross-section from Zones 1 to 7.

n.s.: not significant. *; p<0.05, **: p<0.01

|  | Maximum  principal  stress | Minimum  principal  stress | Equivalent  stress | Maximum  principal  strain | Minimum  principal  strain | Strain  energy  density |
| --- | --- | --- | --- | --- | --- | --- |
| zone 1 | 0.49** | -0.55** | 0.53** | 0.78** | -0.82** | 0.73** |
| zone 2 | 0.52** | -0.58** | 0.54** | 0.70** | -0.74** | 0.71** |
| zone 3 | 0.38* | -0.55** | 0.48** | 0.70** | -0.75** | 0.68** |
| zone 4 | n.s. | -0.44** | 0.39** | 0.51** | -0.66** | 0.59** |
| zone 5 | n.s. | -0.32* | 0.30* | 0.51** | -0.64** | 0.44** |
| zone 6 | n.s. | n.s. | n.s. | 0.48** | -0.62** | 0.39** |
| zone 7 | n.s. | n.s. | n.s. | 0.54** | -0.56** | 0.34* |

Supplementary Table 2. Correlation coefficients of biomechanical parameters and

the reciprocal of the cortical index from Zones 1 to 7.

n.s.: not significant. *; p<0.05, **: p<0.01

|  | Cortical bone thickness/  Area of the cross-section | Cortical bone thickness/  Cortical Index | Area of the cross-section/  Cortical Index |
| --- | --- | --- | --- |
| zone 1 | 0.91** | 0.97** | 0.79** |
| zone 2 | 0.89** | 0.97** | 0.76** |
| zone 3 | 0.87** | 0.96** | 0.74** |
| zone 4 | 0.86** | 0.96** | 0.71** |
| zone 5 | 0.84** | 0.96** | 0.67** |
| zone 6 | 0.83** | 0.96** | 0.65** |
| zone 7 | 0.87** | 0.95** | 0.69** |

Supplementary Table 3. Correlation coefficients of biomechanical parameters and

morphological parameters from Zones 1 to 7. **: p<0.01
